# Using an optimized generative model to infer the progression of complications in type 2 diabetes patients

**DOI:** 10.1186/s12911-022-01915-5

**Published:** 2022-07-01

**Authors:** Xiaoxia Wang, Yifei Lin, Yun Xiong, Suhua Zhang, Yanming He, Yuqing He, Zhikun Zhang, Joseph M. Plasek, Li Zhou, David W. Bates, Chunlei Tang

**Affiliations:** 1 Shanghai Key Laboratory of Data Science, School of Computer Science, Fudan University, Shanghai, 200438 China; 2 College of Computer Science and Engineering, Northwest Normal University, Gansu, 730070 China; 3 West China Hospital of Sichuan University, Sichuan, 610041 China; 4 Department of Kidney Disease, Suzhou Kowloon Hospital, Shanghai Jiao Tong University School of Medicine, Jiangsu, 215021 China; 5 Department of Endocrinology, Yueyang Hospital of Integrated Traditional Chinese Medicine and Western Medicine, Shanghai University of Traditional Chinese Medicine, Shanghai, 200437 China; 6 Institute for Data Industry, School of Economics, Fudan University, Shanghai, 200433 China; 7 Division of General Internal Medicine and Primary Care, Brigham and Women’s Hospital, Harvard Medical School, Boston, MA 02115 USA; 8 Clinical and Quality Analysis, Mass General Brigham, Boston, MA 02145 USA; 9 Division of General Internal Medicine and Primary Care, Brigham and Women’s Hospital, 1620 Tremont Street, Boston, MA 02120 USA

**Keywords:** Computer simulation, Disease progression model, Diabetes mellitus, type 2, Probabilistic generative model, Electronic health records

## Abstract

**Background:**

People live a long time in pre-diabetes/early diabetes without a formal diagnosis or management. Heterogeneity of progression coupled with deficiencies in electronic health records related to incomplete data, discrete events, and irregular event intervals make identification of pre-diabetes and critical points of diabetes progression challenging.

**Methods:**

We utilized longitudinal electronic health records of 9298 patients with type 2 diabetes or prediabetes from 2005 to 2016 from a large regional healthcare delivery network in China. We optimized a generative Markov-Bayesian-based model to generate 5000 synthetic illness trajectories. The synthetic data were manually reviewed by endocrinologists.

**Results:**

We build an optimized generative progression model for type 2 diabetes using anchor information to reduce the number of parameters learning in the third layer of the model from $O\left(N\times W\right)$ to $O\left((N-C)\times W\right)$, where $N$ is the number of clinical findings, $W$ is the number of complications, $C$ is the number of anchors. Based on this model, we infer the relationships between progression stages, the onset of complication categories, and the associated diagnoses during the whole progression of type 2 diabetes using electronic health records.

**Discussion:**

Our findings indicate that 55.3% of single complications and 31.8% of complication patterns could be predicted early and managed appropriately to potentially delay (as it is a progressive disease) or prevented (by lifestyle modifications that keep patient from developing/triggering diabetes in the first place).

**Conclusions:**

The full type 2 diabetes patient trajectories generated by the chronic disease progression model can counter a lack of real-world evidence of desired longitudinal timeframe while facilitating population health management.

## Introduction

Patients with type 2 diabetes usually have few, if any, symptoms initially. Patients’ persist in the prediabetes phase for years; thus, the disease is often undetected until it progresses to a chronic condition as serious complications develop. It is estimated that more than 1 in 3 American adults (~ 88 million) and nearly 36% of the Chinese adult population have prediabetes [1, 2]. When pre-diabetes converting to diabetes and that progression in early stages of diabetes, patients either have insulin resistance where the body still produces insulin but is unable to effectively use insulin, or they don’t produce enough insulin, leading to accumulation of glucose in the bloodstream. Diabetes affects multiple major organs and its most frequent complications include myocardial infarction, stroke, neuropathy, kidney damage, and microvascular events [3–7].

In the medical field, a large amount of data (e.g., laboratory test results, clinical findings, diagnoses, symptoms, and medication treatments) recorded in electronic health record (EHR) systems can facilitate clinical knowledge discovery. However, EHR data has inherent limitations for studying progression of type 2 diabetes from prediabetes to overt diabetes, including its static nature (e.g., family history information, genetic testing results), missing values, and irregularity (e.g., data are recorded at discrete time points with non-equal intervals). Disease progression models (DPM) require substantial domain knowledge on disease stages, vital indicators/ measurements, and insight into the target diseases epidemiology. Watabe et al. [8] employed a hierarchical Bayesian framework to infer the progression level to diabetes based on oral glucose tolerance tests. It is not a true DPM due to not focusing on the slow development of diabetes over time. Marini et al. [9] developed a Dynamic Bayesian Network (DBN) model to simulate of development of several clinical complications of type 1 diabetes. Islam et al. [10] applied a machine learning pipeline to predict future development of type 2 diabetes based on finding an optimal set of risk-factors. This is not the case for our purpose because we aim to use a minimally supervised approach to generate the full trajectories of chronic diseases, which does not require either a training dataset with patient disease stages labeled or domain knowledge that specifies the indicators for stage transitions.

**Fig. 1:**
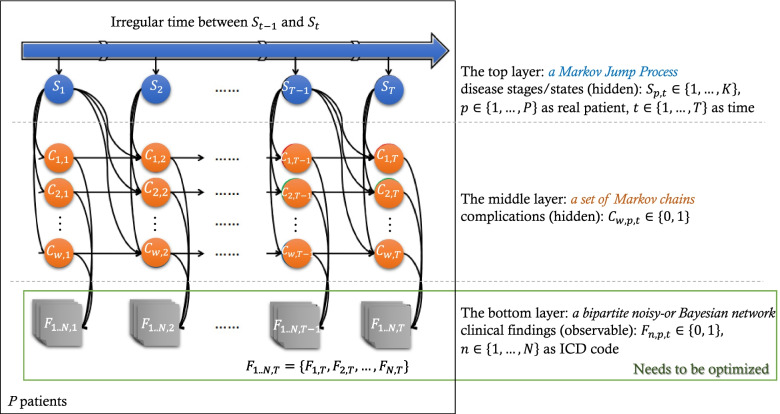
The outline of Wang et al.’s model, where $K$ is the number of disease stages, $M$ is the number of complications, and $N$ is the number ICD codes

Type 2 diabetes is a chronic disease with a progression trajectory that can span 30 + years from pre-diabetes to severe complications and death meaning that most EHR datasets only cover a portion of the relative longitudinal trajectory. In order to cover the full longitudinal trajectory, synthetic data generated using probabilistic generative models can be a suitable proxy. Sukkar et al. [11] used unsupervised hidden Markov models to create a general disease progression model. Wang et al. [12] utilized a three-layer pipeline (Fig. 1) consisting of the Markov Jump Process, Markov Chain, and noisy-or Bayesian network to similarly create an unsupervised disease progression model that infers progression from the onset of comorbidities. In this model, a comorbidity is a disease or syndrome that co-occurs with the target disease. Comorbidities are assumed to be conditionally independent, given the state of the target disease. The bottom layer is a bipartite noisy-or Bayesian network that is used to infer the presence of the comorbidities from the observed clinical findings (e.g., ICD codes). Given a set of ICD codes, Wang et al. assume an observed clinical finding was “activated” by the presence of any of the comorbidities with a certain activation probability; it is also possible that none of the comorbidities is present and the finding was activated by an always-on hidden cause with a leak probability. This layer allows the model to deal with large amounts of clinical findings. The pros of Wang et al.’s model [12] are visible. Its structure is flexible enough to be well suited to the setting, especially for modeling sparse and noisy observations. The model can be used to consider either irregular patient visits or a continuous-time disease progression. The gap in the literature our study focuses on is to improve the time complexity of Wang et al.’s model and adapting the model design to a new population.

In this study, we aim to infer full progression trajectories of type 2 diabetes via synthetic patient generation. Firstly, to learn more efficiently the probabilistic relationships between chronic disease progression stages, complication categories, and clinical diagnoses based on the real incomplete patient records in EHR data sets, we optimize Wang et al.’s model [12] (see Fig. 1). Then we suit the optimized model to build a progression model for type 2 diabetes using a real EHR data set. We further demonstrate the full progression of type 2 diabetes by means of synthetic patients generated by the learned progression model and how the model can facilitate population health management.

## Methods

### Dataset settings

Data was derived from a 17-hospital-based regional healthcare delivery network managed by the local Center for Disease Control (CDC) in Shanghai, China. The data integrates real world electronic health record (EHR) data with “follow-up” data (that was generated from that tracks patient outcomes for those same patients). Data were coded using the International Classification of Diseases—Version 10 (ICD-10-CM) codes. This study was approved by Shanghai CDC’s Institutional Review Board (IRB).

**Fig. 2:**
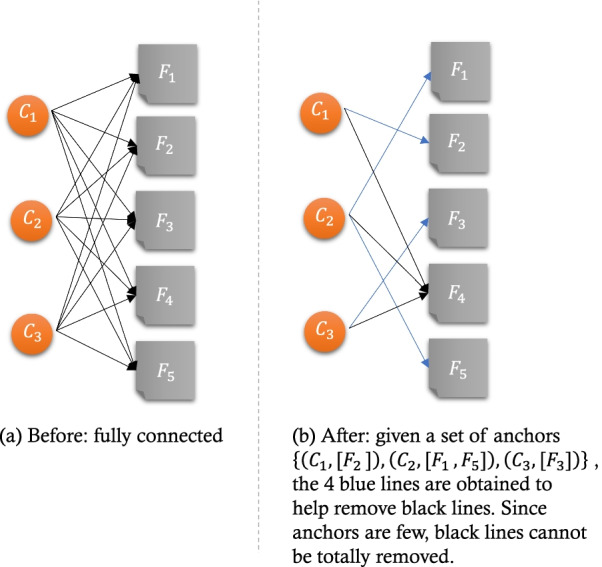
An illustration of before and after optimizing the observable bottom layer

Our dataset consists of 9298 real patients with confirmed type 2 diabetes over an 11-year timespan from January 2005 to January 2016, in which 43.3% (4028/9298) were male, 100% had been hospitalized in the facility at some point during the timespan for any cause, and a 3.9% mortality rate (367/9298, 188 males and 179 females). We retrieved a total of 1311 distinct ICD codes relating to these patients’ comorbidities from the data. We next removed infrequent ICD-10 codes (i.e., 1223 of 1311) that appeared less than 30 times, leaving 88 distinct ICD-10 codes for use in the generative models. Considering that type 2 diabetes progress slowly, we integrated the patient records within 1 year into a time slice as an encounter. Since the model needs to calculate the interval between two adjacent encounters, we excluded patients whose total number of time slices are less than 2 from the data. Figure 2 shows the number of positive observations in each encounter for each real patient, and demonstrates our data are very sparse. Furthermore, note that it is a big challenge to learn a full progression model for type 2 diabetes using data which time span are shorter than the common type 2 diabetes progression.

### Model optimization

The time efficiency of Wang et al.’s is largely dependent on the number of a variety of variables and time slices. More specifically, given 1000 distinct ICD codes (i.e., N) of patients’ clinical findings relating to type 2 diabetes, 10 complications (i.e., W), and 5 disease stages (i.e., K), the number of model parameters is 10,125 calculated by the following formula.

1 $$\begin{aligned} & \left( {K \times K} \right) + 2\left( {K \times W} \right) + \left( {N \times W} \right) \\ & \quad = \left( {5 \times 5} \right) + 2\left( {5 \times 10} \right) + \left( {10 \times 1,000} \right) = 10,125 \\ \end{aligned}$$

The idea of our optimization is to prune (i.e., reduce the number of parameters) the fully connected observable bottom layer. We consulted our medical experts to add some clinical pieces of knowledge. They believe that most ICD-10 codes only belong to one complication relating to type 2 diabetes. We referred to these pieces of medical knowledge as “anchors.” A set of anchors as prior knowledge given by our medical experts is set to a known probability of obtaining a better-interpreted clustering result of the complication group. Due to no recalculation, the model can save most of the time to run and performs excellently. Also, anchors are few enough to make the model unsupervised. In our optimized model, we considered the observed data as clinical diagnoses (i.e. ${F}_{w,p,t}$ are ICD-10 codes), and the third layer as complications related to a group of corresponding clinical diagnosis (i.e. ${C}_{n,p,t}$ are complications). Figure 2a shows that the initial layer is fully connected. As shown in Fig. 2b, given a set of anchors $\{\left({C}_{1},\left[{F}_{2}\right]\right), \left({C}_{2},\left[{F}_{1},{F}_{5}\right]\right), ({C}_{3},\left[{F}_{3}\right])\}$ corresponding to a set of complications $\{{C}_{1},{C}_{2}{,C}_{3}\}$ and a set of clinical diagnoses $\{{F}_{1},{F}_{2}, {F}_{3},{F}_{4}, {F}_{5}\}$. The anchors help strengthen any possible links with high probability based on prior knowledge (i.e., blue lines). Using anchor $\left({C}_{2},\left[{F}_{1},{F}_{5}\right]\right)$, which is means clinical diagnoses ${F}_{1}$ and ${F}_{5}$ should be clustered into complication ${C}_{2}$, we prune all other links to ${F}_{1}$ and ${F}_{5}$, and only links ${{C}_{2}\to F}_{1}$ and ${C}_{2}{\to F}_{5}$ are left. Since few anchors exist, it is impossible to determine all possible links with low probability, so that some necessary lines may remain. For example, for a clinical diagnosis ${F}_{4}$, none of the anchors helped at removing the black lines, and it remains a fully connected with all three complications ${C}_{1},{C}_{2}, {C}_{3}$.

In addition, we considered an alternate to improve efficiency; that is, to give an enlarged time granularity and reduce the number of time slices. We integrate the patient records within 1 year into a single time slice. This is a mild assumption in our case because generally type II diabetes is a common chronic disease with a slow progression.

### Data analysis and statistics

As a comparison, we trained the original Wang et al*.* model on our data and compared the run time against our optimized J Med Internet Res J Med Internet Res model using a computer with 2 GeForce GTX1080 Yi 11G cards.

We first change the following main parameters of our optimized model to suit type 2 diabetes and learn a generative unsupervised progression model, where disease stages/states and onset of diabetes complications were hidden variables. Type 2 diabetes’ disease stages/states were specified as 5 (i.e., $K=5$) by referencing the diabetes complications severity index (DCSI) [13]. With two medical experts’ help and literature reviewing [14], we set the number of complication categories to 12 (see Table 1), namely $W=12$. Note that $N=88$ because there are 88 distinct ICD-10 codes after data preprocessing.

**Table 1:** Anchor settings

Serial number	Complication	Comorbidities (ICD-10 code-based anchors)
1*	Diabetes	**E11.9** (Diabetes without complications)
2	Acute complications	**E11.0** (Diabetes with coma), **E11.1** (Diabetes with ketoacidosis), K81 (Cholecystitis), J20 (Bronchitis)
3	Cardiovascular	I25 (Chronic ischemic heart disease), I10 (Hypertension)
4	Nephropathy	**E11.2** (Diabetes with renal complications), N18 (Chronic nephrosis)
5	Ophthalmopathy	**E11.3** (Diabetes with ophthalmic complications), H26.9 (Cataracts, unspecified)
6	Peripheral vascular	**E11.5** (Diabetes with peripheral circulatory complications), I83 (Varicose vein of lower extremity)
7	Cerebrovascular	I63 (Cerebral infarction), G45 (Transient cerebral ischemic attacks)
8	Neuropathy	**E11.4** (Diabetes with neurological complications), G63.2 (diabetic polyneuropathy)
9	Metabolic complications	**E11.6** (Diabetes with other specified complications), E78 (Lipidemia)
10	Tumor	Z51.1 (Chemotherapy session for neoplasm), C34 (Malignant neoplasm of bronchus), C16 (Malignant neoplasm of stomach)
11	Musculoskeletal	M48 (Spondylodynia), M13 (Arthritis), M81 (Osteoporosis)
12	Autoimmune diseases	K52 (Gastroenteritis), E04 (Goiter), J45 (Asthma)

Bold is any diabetes-related ICD code

*Diabetes itself needs an anchor to deal with the case of “no complications.”

Then using the learned progression model, we infer the personal progression trajectories of real patients based on the history medical records. For comparison purposes, our medical experts helped to retrieve two additional representative cases with different development rates. We used the maximum a posteriori (MAP) inference that gives a point estimate by maximizing a posterior probability, the conventional approach in Bayesian statistics to infer progression trajectories based on existing evidence.

We next followed Wang et al.’s assumption to generate synthetic patient with full progression trajectories of type 2 diabetes by initializing each patient to timestamp 0 in state I, and using our optimized model to generate the patient’s subsequent stages and the complication onsets corresponding to those stages until the last stage.

## Results

**Fig. 3:**
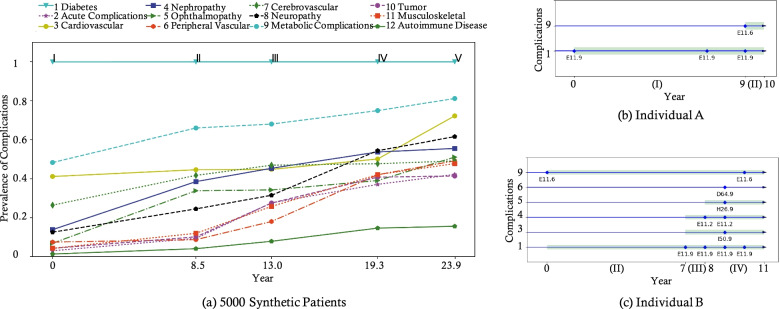
A Comparison of complications of 5000 virtual patients learned by our optimized model (**a**) as well as two representative patients retrieved by our medical experts (**b** & **c**). Note that for (**a**), we first use 35,210 encounters with 64,383 positive observations to learn our generative model. We next use this learned generative model to generate 5000 (that is a specified number) synthetic patient trajectories. Thus, 8.5, 13.0 19.3, 23.9, etc., were the mean progression years at stages I to V, respectively

A total of 35,210 encounters with 64,383 positive observations were input into our optimized model to generate 5000 (that is a specified number) synthetic patient trajectories. We averaged over these 5000 patient records and computed the average holding time for each state, as summarized in Fig. 3a. Figure 3a covers a 23.9-year progression path of type 2 diabetes, which is approximately double the timespan of the available data (i.e., 11 years). Specifically, the average duration of each disease stage/state and the prevalence of each complication at different stages are computed.

The time to run the original Wang et al.’s model based on our data was more than 10 days (each iteration needs about one hour, and each execution requires thousands of iterations). In contrast, our optimized model runs to converge within 8 days. Anchors are few enough (12 anchors) that we consider the model to be minimally supervised.

Figure 3b and c illustrate the inferred maximum posterior probability results for the two real individuals’ personal disease progression trajectories. For individual A, the MAP inference only infers one possible complication as “complication 9: metabolic complications;” and our model also gives such a finding and further predicts that the progression will move to the next stage (i.e., stage II) in the ninth year. Individual B is the case of rapid deterioration. While having no ICD-10 codes related to diabetes directly (i.e., E11.9), our model determines the onset of diabetes because of finding some specified complications (e.g., E11.6); in clinical practice, these complications can be found after people with type 2 diabetes diagnosed. Our model assigns state II at the beginning of this progression trajectory and predicts that nephropathy (i.e., E11.2) and cardiovascular (i.e., I50.9) may show up after 7 years. At that time, individual B’s transition from state II to state III is highlighted. The model also points out that ophthalmopathy (i.e., H26.9) will quickly follow developing state III in the next year. Then the progression path will subsequently move into state 4 under a life-threatening condition. Even though anemia (i.e., D64.9) belongs to peripheral vascular complications, yet there is no evidence to support it—our model indicates its activation probability is slightly small.

Figure 3 indicates possibilities to help understand diabetes and associated complications. We wonder if any retrospective possibilities can be generated and evaluated. With illness trajectories viewed by our medical experts, we generated Tables 2 and 3 to show the transition from a later state to an earlier state based on the last state (i.e., stage V). Table 2 is to calculate all probabilities of showing before stage V based on one single complication, and Table 2 is on two commonly shown complication patterns that reflects the co-occurrence of several complications (i.e., such as pattern [3, 7, 8] and pattern [4, 5, 6]). For example, complication 2 as “acute complications” occurred in state V has 2096 virtual patients accounting for 41.9% (2096/5000), and is extremely low (161 virtual patients accounting for 7.7%, 161/2096) compared with stage V. We followed our experts’ opinions to keep; for example, complication 6 as “peripheral vascular” can either occur as a single one or in with other complications such as [6] (14.6%), [4, 6] (2.8%), [5, 6] (2.5%), [4, 5, 6] (0%) in stage I (compared with stage V). Note that relevant clinical meanings amongst the patterns are not the focus of this study. The table aims to demonstrate the information and knowledge among the entire disease progression via virtual patients, which can facilitate population health management.

**Table 2:** Single complication statistics of transition from a later state to an earlier state given by 5 K generated patients

Complication stage		2 Acute complications	3 Cardiovascular	4 Nephropathy	5 Ophthalmopathy	6 Peripheral vascular	7 Cerebrovascular	8 Neuropathy	9 Metabolic complications	10 Tumor	11 Musculoskeletal	12 Autoimmune diseases
Number	V	2096 41.9%	3622 72.4%	2723 54.5%	2600 52.0%	2430 48.6%	2477 49.5%	3110 62.2%	4102 82.0%	2085 41.7%	2381 47.6%	811 16.2%
Probability												
Later to earlier	V → IV	1834 87.5%	2506 69.2%	2617 96.1%	2002 77.0%	2085 85.8%	2390 96.5%	2746 88.3%	3782 92.2%	2062 98.9%	2114 88.8%	753 92.8%
	V → III	1352 64.5%	2238 61.8%	2176 79.9%	1750 67.3%	902 37.1%	2356 95.1%	1567 50.4%	3401 82.9%	1420 68.1%	1300 54.6%	375 46.2%
	V → II	461 22.0%	2228 61.5%	1822 66.9%	1729 66.5%	423 17.4%	2093 84.5%	1213 39.0%	3319 80.9%	544 26.1%	600 25.2%	182 22.4%
	V → I	161 7.7%	2032 56.1%	634 23.3%	335 12.9%	372 15.3%	1375 55.5%	650 20.9%	2420 59.0%	229 11.0%	226 9.5%	59 7.3%

All probabilities are based on stage V

**Table 3:** Two commonly complication patterns’ statistics of transition from a later state to an earlier state given by 5 K generated patients

Complication pattern stage		[3 Cardiovascular, 7 cerebrovascular, 8 neuropathy]							[4 Nephropathy, 5 ophthalmopathy, 6 peripheral vascular]						
Number	V	1132							717						
Probability		22.6% (1132/5000)							14.3% (717/5000)						
Later to earlier (%)		[3]	[7]	[8]	[3, 7]	[3, 8]	[7, 8]	[3, 7, 8]	[4]	[5]	[6]	[4, 5]	[4, 6]	[5, 6]	[4, 5, 6]
	V → IV	69.2	95.7	88.1	66.3	61.0	84.2	58.5	96.8	75.9	85.2	73.5	82.7	63.9	62.1
	V → III	61.7	94.3	49.7	57.9	30.4	46.7	28.4	81.3	66.4	37.2	54.3	30.7	25.2	21.3
	V → II	61.2	82.4	39.2	49.6	23.2	31.9	18.5	68.9	66.1	17.4	44.2	12.3	11.2	7.9
	V → I	55.6	55.6	20.9	30.0	11.2	11.0	6.0	22.2	14.2	14.6	3.2	2.8	2.5	0

All probabilities are based on stage V

## Discussion

Our main finding was that it was feasible to optimize a minimally supervised generative model to simulate synthetic patient trajectories from EHR data focused on the progression of complications in prediabetic patients and patients diagnosed with type 2 diabetes. Our proposed model is built upon a model proposed by Wang et al. [12], which focused on modeling a chronic obstructive pulmonary disease (COPD) patient cohort. The model we modified, enhanced and employed for learning disease progression is scalable; it can comfortably accommodate new sources of data with clinical findings or outcomes. For instance, the lists of medications prescribed or procedures performed, the distribution over the initial disease stages/states (e.g., a function of age, gender, and family history), and patient’s social-behavioral history and habits (e.g., smoking, alcohol use) can be included as a supplement. With progression trajectories depicted by our optimized model, we can obtain some insights. These include, but are not limited to, what disease stages/states the patient traverses, how rapidly the disease develops, which complications can be found, and how long specified complications give the stage transition. This model is suitable for finding relationships between disease progression and complication patterns. Therefore, modification of parameter values, number of complication groups, number of target disease progression stages, etc., can be adopted to modeling other chronic disease (e.g., obesity and metabolic diseases) and their complications.

Previously, the idea of “synthetic patient simulations” were utilized for educational purposes (e.g., pre- and post-registration health care professional education [15]). In this way, learning of disease progression can also achieve the purpose of teaching patients or health professionals, and even healthy non-patients included. This is because; there exists no the completely GOLD criteria for partitioning type 2 diabetes stages/states, according to the American Diabetic Association guidelines. Take the DCSI (diabetes complications severity index) as an example; while having a higher citation count, the DCSI remains a reference designed by Glasheen et al. [13] rather than an indicator used widely in clinical practice. Our study may offer some evidence to help evaluate such indicators and then to facilitate uniform clinical guidelines. According to Tables 1 and 3, for example, our findings indicate that 55.3% of single complications and 31.8% of complication patterns could be predicted early and managed appropriately to potentially delay (as it is a progressive disease) or prevented (by lifestyle modifications that keep patient from developing/triggering diabetes in the first place). Figure 3a indicates some interesting clinical insights. First, we found that metabolic complications showed up most frequently, which is throughout the entire progression of type 2 diabetes starting at the very beginning. In contrast, autoimmune diseases were the most infrequent. Second, the top 3 complications, are cardiovascular, cerebrovascular, and metabolic complications, imply that it is necessary to recommend laboratory tests regularly for prediabetic patients. Third, nephropathy and ophthalmopathy both are microvascular complications and may be useful to study in the early stages, especially in the transition from stage I to II. Last, acute complications, as well as nephropathy, peripheral vascular, and musculoskeletal complications present an increased risk after stage II. Figure 3a indicates that autoimmune diseases are a relatively rare complication. This is expected as existing knowledge about type 2 diabetes; namely, developing type 2 diabetes doesn’t mean that the body cannot produce insulin (such like type I diabetes, which is an autoimmune disease; the immune system attacks the pancreas, so it can’t make insulin) [14]. The fact that the body is often unable to effectively use insulin to accumulate glucose in the bloodstream [3, 14]. Nevertheless, these prior research have found evidence that insulin resistance may be the result of immune system cells attacking the body’s tissues rather than just a metabolic disorder , which warrants further investigation.

Although having constrained the continuous-time Markov model to allow only forward transitions, synthetic patient simulations without disease stages/states of absence can help better understand the evolution of chronic illnesses in reverse. Therefore, we can try to conduct research in population medicine through this research and take population health management as pro-active management to improve health and resolve health disparities relating to diabetes and prediabetes.

The main limitation of our study is that this work was based on a single chronic disease, type 2 diabetes, and thus our results may not generalize to other chronic conditions. In addition, our study only used one kind of data, ICD-10 codes, and therefore might limit clinical insights on population medicine which could include other sources of data, e.g., population-level data, monitoring, surveillance data, and social media data..

## Conclusions

In this study, we employed the generative nature of Wang et al.’s model [12] to infer the progression of complications in type 2 diabetes patients. After adding 12 anchors based on prior domain knowledge, the model’s fully connected observable bottom layer is pruned to reduce runtime significantly. These anchors only use few manual efforts so that our model is minimally supervised. Our main findings are (1) that a generative model can help solve incomplete and/or insufficient data problems to better understand the whole trajectories of lowly and long progression of chronic disease, and (2) it is feasible to facilitate population health management (e.g., prediabetes) as a statistical retrospect or prediction of synthetic patient trajectories.

## Data Availability

Our research data is unavailable for access because it is confidential. According to the HIPAA standard, it would be cost-prohibitive to sufficiently de-identify such a large corpus of clinical documents to remove all patient identifying data. Yun Xiong could be contacted if someone wants to request the data from this study.
